# Prevention and Control of COVID-19 in Italian Prisons: Stringent Measures and Unintended Consequences

**DOI:** 10.3389/fpubh.2020.559135

**Published:** 2020-09-17

**Authors:** Lara Tavoschi, Roberto Monarca, Ruggero Giuliani, Alessio Saponaro, Stefano Petrella, Roberto Ranieri, Filipa Alves da Costa, Carina Ferreira-Borges, Linda Montanari

**Affiliations:** ^1^Department of Translational Research and New Technologies in Medicine and Surgery, University of Pisa, Pisa, Italy; ^2^Health Without Barriers, The European Federation for Prison Health, Viterbo, Italy; ^3^Department of Infectious Diseases, Viterbo Local Health Unit, Viterbo, Italy; ^4^Infectious Diseases Service, Penitentiary Health System, San Paolo University Hospital, Milan, Italy; ^5^Emilia Romagna Region, Bologna, Italy; ^6^Penitentiary Health System, Modena Local Health Unit, Modena, Italy; ^7^WHO European Office for Prevention and Control of Noncommunicable Diseases (NCD Office), Moscow, Russia; ^8^Public Health Unit, European Monitoring Centre for Drugs and Drug Addiction, Lisbon, Portugal

**Keywords:** COVID-19, pandemic, prison, Italy, prevention and control, people who use drugs (PWUD)

## Introduction

The need to integrate prisons and other custodial settings in the comprehensive response to COVID-19 epidemic was recently advocated (1) and WHO as recently issued guidance to support members states in this direction (2).

The COVID-19 pandemic has been particularly dramatic in Italy, one of the first countries to be affected in Europe, with more than 200,000 cases reported as of 22/4/2020 (3). Within the country, the northern regions, including Lombardy and Emilia Romagna, were the epicenter of the epidemic and massive efforts were put in place to contain its spread. The custodial system has been part of this wider endeavor, as prison healthcare services are managed by Ministry of Health in Italy, although with differences across regions due to healthcare decentralization.

## The Prison Setting

Prisons are settings of higher risk for COVID-19 infections as confined conditions, especially in a context of overcrowding, are one of the biggest challenges for controlling the spread of the infection. Italy is the third country in Europe per prison density with an occupational rate of 120% for 61,230 prison population at 29/2/2020 (4). People in prison are more vulnerable to COVID-19 because of their underlying health conditions with disproportionally higher rates of acute and chronic physical and mental illnesses, including cardiovascular diseases, diabetes and chronic respiratory diseases, and frequently facing greater exposure to risks such as smoking, poor hygiene and weaker immune defense to stress (5).

## Response Measures in Prison Settings

Avoiding COVID-19 spread into the custodial system is the primary objective of an effective strategy tailored to prisons. In the early stage of the epidemic a rapid scale-up of prevention and control measures was implemented in the northern regions in close coordination with relevant health authorities. Triage and syndromic screening were set-up for all individuals entering prison premises, including staff, visitors and incoming detainees. Dedicated areas for triaging were identified and in 77% (151/197^1^) of existing institutions temporary tensile-structures were put in place. Collection of biological samples and access to laboratory facilities was ensured as per standard community protocols. Areas for medical isolation (dedicated wings, single detention rooms, COVID-19 prison hub) of close contacts/suspects/confirmed cases were designated and provided with adequate protective measures, in order to minimize risks of transmission within prison and to allow for management of mild COVID-19 cases. Severe cases were transferred to referral tertiary hospitals in the community. Adequate supply of personal protective equipment and disinfectants was managed in collaboration with Civil Protection Agency. As the epidemic spreads across the country, national guidance was also issued (6).

## Additional Measures

The Ministry of Justice early on in the epidemic response issued organizational recommendations and stringent limitation on admission to prison premises, in particular restricting access to essential staff and banning visitors including relatives (7). The measure was deemed necessary to minimize COVID-19 introduction risks, and swiftly implemented. To mitigate its impact, alternatives to face-to-face visits were gradually implemented. Yet, when enacted, the measure sparked unrest across the whole country, with serious events occurring in some institutions. In Modena and Milan prisons people assaulted pharmacies ingesting large quantity of opioids used to treat drug addictions. Nine persons died in 1 day in the Modena's prison (8), although was ongoing at the time of writing. Like in many other countries, people with drug use disorders are overrepresented in prison, with 28% of the entire Italian prison population falling in this category and 34% being incarcerated for drug related crimes at 31/12/2018 (4, 9). Alternative measures to incarcerations (house arrest for up to 5,000 individuals) currently implemented within the COVID-19 response framework to reduce the number of inmates (10), might largely involve the sub-groups of drug users and people incarcerated for drug related crimes (9). Therefore, while COVID-19 prevention remains a primary concern, appropriate management of addiction, including linkage to community drug and social services, is necessary to respond to released individuals' health needs.

## Current Assessment of Impact

Still, the data currently available at this early stage suggest that the introduction of prevention and control measures had a positive impact on the spread of COVID-19 into and within the Italian prison system. More than 8-week into the epidemic with thousands of cases reported, only few cases occurred in prison. In Lombardy (11) and in Emilia-Romagna regions, where prison services swiftly implemented thorough prevention and control protocols, respectively 19 and 14 COVID-19 cases were reported, including one death, as of 22/4/2020.

## Conclusions

While COVID-19 cases in the prison system are unavoidable, heightened attention along with stringent and comprehensive measures are needed when country-wide lockdown measures are relaxed. The COVID-19 pandemic calls on us to fulfill the principle “prison health is public health” in order to protect the well-being of people in prison and their community, uphold equity and avoid serious organizational, security and safety dangers resulting from outbreaks occurring in this setting.

## Author Contributions

LT and LM conceived the manuscript. All authors contributed to manuscript drafting, read, and approved the final version.

## Conflict of Interest

The authors declare that the research was conducted in the absence of any commercial or financial relationships that could be construed as a potential conflict of interest.

## Footnotes

^1^As of 4th of April 2020.
